# Precision public health alliances as a model and method for community engagement

**DOI:** 10.3389/fpubh.2025.1617776

**Published:** 2025-08-21

**Authors:** Margaret L. McGladrey, Mary Elizabeth Lacy, Kristen McQuerry, Rachel Hogg-Graham, Svetla Slavova, Emily R. Clear, Kory Heier, Caitline Phan, Megan Hall, Ryan Parker, Mindy Keeton, Jennifer Sword, Thomas Ard, Charbel Salem

**Affiliations:** ^1^College of Public Health, University of Kentucky, Lexington, KY, United States; ^2^King’s Daughters Medical Center, University of Kentucky, Ashland, KY, United States

**Keywords:** precision public health, social determinants of health, community-based participatory research, community health planning, community networks, electronic health records

## Abstract

Using precision analytics approaches with population health data helps identify localized patterns of social determinants and comorbidities, supporting the design of tailored interventions. The University of Kentucky College of Public Health (UKCPH) and UK King’s Daughters (UKKD) have partnered to create a Precision Public Health Alliance (PPHA) applying precision analytics to UKKD electronic health records (EHR) as well as secondary datasets to map social, demographic, and clinical comorbidity factors onto colorectal cancer (CRC) screening data in UKKD’s rural service area (the northeastern Kentucky counties of Boyd, Carter, Greenup, and Lawrence and southeast Ohio county of Lawrence). In addition to UKKD and UKCPH clinicians and researchers, PPHA includes a community-based Action Team of local social services, behavioral health, and public health agencies and Cooperative Extension agents responsible for translating findings into quality improvement priorities. In Fall 2024, UKKD and UKCPH developed a statistical analysis plan to examine the interplay of social, geographic, and clinical factors associated with CRC screening rates, which is a UKKD quality improvement priority. Findings from this analysis of EHR and secondary county-level data and a summary of UKKD’s existing quality improvement efforts related to CRC screening were presented to the Action Team in March 2025. The first meeting of the Action Team demonstrated initial proof-of-concept that PPHA approaches can catalyze community-based practice improvements. Immediately after the meeting with UKCPH support, the local health department and community mental health center pursued adoption of UKKD stool-based screening strategies and UKKD created a simple workflow for receiving referrals from community-based partners. This community case study contributes to community engagement with initial findings and best practices for PPHA of healthcare systems, local public health departments, and social services agencies to analyze population health trends in order to target quality improvement needs and co-design place-based, cross-sector strategies to address them with evidence-based practices. This replicable PPHA pilot in rural Kentucky and Ohio is an innovative method and model for how to engage communities most effectively in identifying and taking action to improve their chief health concerns.

## 1 Introduction

Community engagement in efforts to improve health and social outcomes is increasingly prioritized to develop and sustain public health education and promotion interventions. However, community-engaged public health practice and research is undermined by imprecise definitions of what constitutes a “community” and the lack of consensus on methods and best practices (1–3). Stronger community engagement models and methods are needed to account for contextual variations in social determinants of health (SDOH), as many non-medical factors contribute to a person’s health, including health behaviors, economic policies and systems, and the environment (4–6). Poor health outcomes aggregate across the U.S. and the world in areas with the highest levels of social vulnerability and inequality (5, 7–9). Therefore, one-size-fits-all approaches are ineffective in tailoring and implementing interventions that are place-based and person-centered and that account for social risk factors and structural inequalities (10–13).

Unfortunately, health and social services stakeholders often do not have the local-level data and infrastructure needed to tailor responses to their communities’ unique configurations of SDOH (14). In response, healthcare and public health systems have intensified efforts to enhance the use of electronic data standards and data sharing; however, information technology capacity and interoperability remain limited due to varying terms of data use agreements as well as privacy and proprietary concerns (14, 15). Combining precision medicine with health systems approaches, precision public health (PPH) has been proposed as a solution that harvests real-world “organic data” collected routinely, automatically, and in real-time (e.g., genomics, clinical, social, patient-generated, environmental, demographic) to assess and target place-based patterns of disease burden, with the goal of delivering “the right intervention to the right population at the right time” (16, 17). Yet, technically oriented PPH definitions and practices that are focused primarily on genomics run the risk of overlooking the human factors of teaming behaviors, social norms and determinants of health, political will, and systemic investments in prevention that ultimately determine the impact and utility of PPH (17–21).

Additionally, research questions involving real-world data such as electronic health records (EHR) are inherently cross-disciplinary, requiring expertise in database management, data abstraction, and statistical modeling. Real-world data systems are vast and variable, and extracting meaningful information demands careful attention to data quality, linkage, and completeness (22). As such, PPH is best approached through team science, where biostatisticians and epidemiologists work collaboratively with clinicians, community-based partners, and health services researchers (23, 24). Furthermore, PPH benefits from the integration of participatory and implementation science methods to translate analytics into quality improvement action that builds learning public health systems (17, 18, 21, 25–27). This community case study aims to improve upon current PPH approaches by sharing the results of rigorous EHR analytics with multidisciplinary clinical and social practitioners to form a cross-sector alliance around data-driven consensus on localized health improvement goals.

## 2 Materials and methods

### 2.1 Community case study solution

This pilot *PPH alliance (PPHA)* approach to community engagement in rural northeastern Kentucky (KY) and southeast Ohio leverages existing clinical-community-academic partnership capacity in population-level data analysis and implementation science methods to build and act on a shared understanding of localized sociodemographic and comorbidity patterns. In contrast to more technically oriented PPH projects, the alliance model is designed to facilitate participatory intervention identification and co-design by cross-sector partners with stakes in improving community health. Across KY’s 120 counties and within its rural regions, substantial variation exists in access to health and social services, geographic features, environmental conditions, educational attainment, community-based organizations, school systems, demography, and regional affiliations. Considering this variation and KY’s burden of poor health outcomes and unmet social needs, KY is an ideal setting to assess the PPHA as a community engagement model and method. The University of Kentucky College of Public Health (UKCPH) and the UK King’s Daughters (UKKD) medical center launched the PPHA pilot funded from July 2024–June 2025 by the UK Provost’s Office to examine the interplay between social and clinical factors that underpin public health challenges specific to the region and populations served by UKKD.

### 2.2 Context

Health outcomes in KY are among the worst in the U.S. In 2023, KY was ranked in the bottom quartile (41st) of health outcomes and consistently ranks among the 10 unhealthiest states suffering from high rates of premature death (44th), obesity (40th), multiple chronic conditions (49th), and mental distress (28th) (28, 29). This gap is even more pronounced in rural regions away from the state’s urban population centers (30, 31). Limited availability of primary care professionals, increased number of uninsured residents, and long distances to clinics compound the difficulties in effectively addressing and managing health conditions in rural regions. In addition to poor health outcomes, KY experiences some of the highest rates of unmet social needs compared with national averages. While approximately 12% of the U.S. population lives in poverty (32), KY’s overall rate is 16.5% and increases to 20.7% in rural communities (33).

Offering comprehensive patient and ambulatory care services for approximately 125 years, UKKD is the cornerstone of healthcare in mostly rural northeastern KY (serving some surrounding Ohio counties) and has accumulated a longitudinal EHR repository since the integration of the EHR system (EPIC) in 2008. UKKD captures consistent and comprehensive data from its stable population base, overcoming typical shortcomings of EHRs, such as data loss as members move in and out of the system. The UKCPH team contributes both analytical and intervention expertise to the PPHA. Combining expertise in examining population trends with methodological support using EHR data, the Biostatistics Consulting & Interdisciplinary Research Collaboration Lab (Biostat CIRCL) provides UKCPH with a team of biostatisticians who specialize in applying rigorous statistical methodology to the complex challenges of interdisciplinary biomedical research. Biostat CIRCL’s mission is to foster strong research partnerships that enable data-driven discoveries and position biostatisticians as essential members of scientific teams (34). Other UKCPH faculty offer capacity and experience with engaging clinicians, researchers, and community-based partners in interpreting population health trends to tailor data-driven prevention and control interventions. Established UKCPH quality improvement collaborations with these cross-sector community-based agencies in the UKKD service area are the foundation of the alliance model.

### 2.3 Human subjects

A fully executed data use agreement between UK and UKKD authorized the secure transfer and use of EHR data for this study. Institutional Review Board (IRB) approval (IRB #99981) was obtained from UK in January 2025. UKKD executed a reliance agreement for UK to be the IRB of record. UKKD provided a limited, de-identified dataset via Excel spreadsheets shared through a secure FTP site, accompanied by a comprehensive data dictionary. To protect patient privacy, all data are stored at the UK Center for Clinical and Translational Sciences Enterprise Data Center, managed by UKKD information technology personnel. Researchers based outside the covered entity access the data exclusively through a secure virtual machine, ensuring protected health information remains within a HIPAA-compliant environment.

### 2.4 Data collection, processing, and analysis

From July–November 2024, the UKKD clinical and population health teams in collaboration with UKCPH identified increasing colorectal cancer (CRC) screening in Boyd, Carter, Greenup, and Lawrence Counties (KY) and Lawrence (OH) as a UKKD priority quality improvement goal that could serve as a pilot of the PPHA approach. CRC rates in KY are among the highest in the country. The U.S. Preventive Services Task Force (2021) recommends CRC screening in adults aged 45–49 years (Grade B recommendation) and in adults aged 50–75 years (Grade A recommendation). We quantified UKKD performance on the CRC screening electronic clinical quality measures (eCQMs). The eCQM for CRC screening quantifies the percentage of patients aged 45–75 who have one or more CRC screenings in the given time frame. The initial project was designed as a retrospective observational study to identify social and geo-location factors associated with CRC screening completion between November 2023 and November 2024. The target population included patients aged 46–75 who had at least one clinical visit during the measurement period. Patients with a history of CRC or total colectomy, or those receiving palliative care or meeting frailty/dementia criteria, were excluded.

Secondary data sources were used to characterize county-level demographic and clinical characteristics of UKKD service counties compared to state- and national-level data. Data sources used include the U.S. Department of Commerce, U.S. Census Bureau 2022 American Community Survey (ACS) (43), the Mapping Medicare Disparities (MMD) (44) Tool (Centers for Medicare & Medicaid Services (CMS), Office of Minority Health, 2020), the University of Wisconsin Population Health Institute County Health Rankings (2024) (45), National Cancer Institute State Cancer Profiles (2021) (46), and the Kentucky Behavioral Risk Factor Survey (2021). ACS is a nationwide survey designed to provide communities with reliable and timely social, economic, housing, and demographic data every year. The ACS has an annual sample size of about 3.5 million addresses. Variables extracted for this analysis include population, median age, and percent living in poverty. The MMD Tool is an interactive visualization tool centered around a state and county-level map of the U.S. It allows users to easily identify the geographic disparities in utilization and outcome variables in the Medicare population. The data used in the Population View are based on CMS administrative enrollment and claims data for Medicare beneficiaries enrolled in the fee-for-service program (47). These data are available from the CMS Chronic Condition Data Warehouse, a database with 100 percent of the Medicare enrollment and fee-for-service claims data. Variables extracted for this analysis include preventable hospitalizations for acute and chronic conditions per 100,000 Medicare beneficiaries. Preventable hospitalization rates are based on 100 percent of inpatient claims. The University of Wisconsin Population Health Institute and the Robert Wood Johnson Foundation’s County Health Rankings provide information on how individuals in one county compare to individuals in other counties on a range of factors that determine health, unemployment, education, community safety, diet, and exercise. National Cancer Institute State Cancer Profiles include age-adjusted incidence rates by cancer site nationally, statewide, and at the county-level; age-adjustment is to the 2000 US Standard Population. Data extracted for this analysis includes colorectal cancer incidence. The Kentucky Behavioral Risk Factor Survey 2021 Annual Report was used to estimate CRC prevalence nationally, statewide, and at the county-level. A Statistical Analysis Plan (SAP) was developed by UKCPH and included descriptive and graphical summaries of sociodemographic and clinical factors stratified by CRC screening status (see Supplementary material for SAP template).

After completing the CRC SAP, UKCPH and UKKD invited established partners from local health departments, the community mental health center, and social services agencies serving the same region as UKKD to the inaugural Action Team meeting in March 2025. In the PPHA model, the role of the Action Team is to develop strategies to improve population health through targeted cross-system service coordination efforts based on analytical findings about which populations do and do not receive prevention services. During this inaugural meeting, the UKCPH team presented the results described in Section 3.1 and asked the Action Team members to identify touchpoints in their service arrays that could be leveraged to improve CRC screening rates through cross-sector collaborative action.

## 3 Results

### 3.1 Secondary and EHR analytical findings

Table 1 describes select population characteristics among UKKD service counties as compared to KY statewide estimates and U.S. national estimates. UKKD service counties included a five-county region along the KY-Ohio border that has an estimated population of 208,201 individuals. The median age in UKKD service counties was 43.2 years compared to 39.4 in KY and 39.0 nationally. The percentage of people living in poverty was 19.4% in UKKD services counties versus 16.3% in KY and 12.6% nationally. Rates of preventable hospitalizations (both chronic and acute), prevalence of hypertension, and age-adjusted CRC incidence were all higher in UKKD service counties than in KY and nationally, diabetes prevalence was similar among UKKD service counties and KY statewide estimates, but both were higher than national. The information in Table 1 is included to underscore the fact that there is a need in this service area to improve access to and uptake of primary care services.

**Table 1:** Comparison of service area to the state and nation.

Relevant variables	UKKD service counties	Kentucky	United States
Population	208,201	4,512,310	333,287,562
Age (median)	43.2	39.4	39.0
% in Poverty	19.4	16.3	12.6
Preventable hospitalizations^b^, chronic^a^	3262.8	2325.0	1878.9
Preventable hospitalization^b^, acute^a^	1412.2	1282.7	919.0
Diabetes, (%)	12.0	12.0	10.0
Hypertension, (%)	49.9	40.9	32.3
Colorectal cancer incidence^a^	53.7	45.9	36.4

UKKD Service Counties include Boyd County, KY; Greenup County, KY; Carter County, KY; Lawrence County, KY; Lawrence County, OH. Reported values come from the Mapping Medicare Disparities (MMD) Tool [Centers for Medicare & Medicaid Services (CMS), Office of Minority Health, 2020] database.

a Presented per 100,000 Medicare beneficiaries.

b Preventable hospitalizations capture encounters that could have been avoided with timely and effective primary care services (40–42).

**Figure 1:**
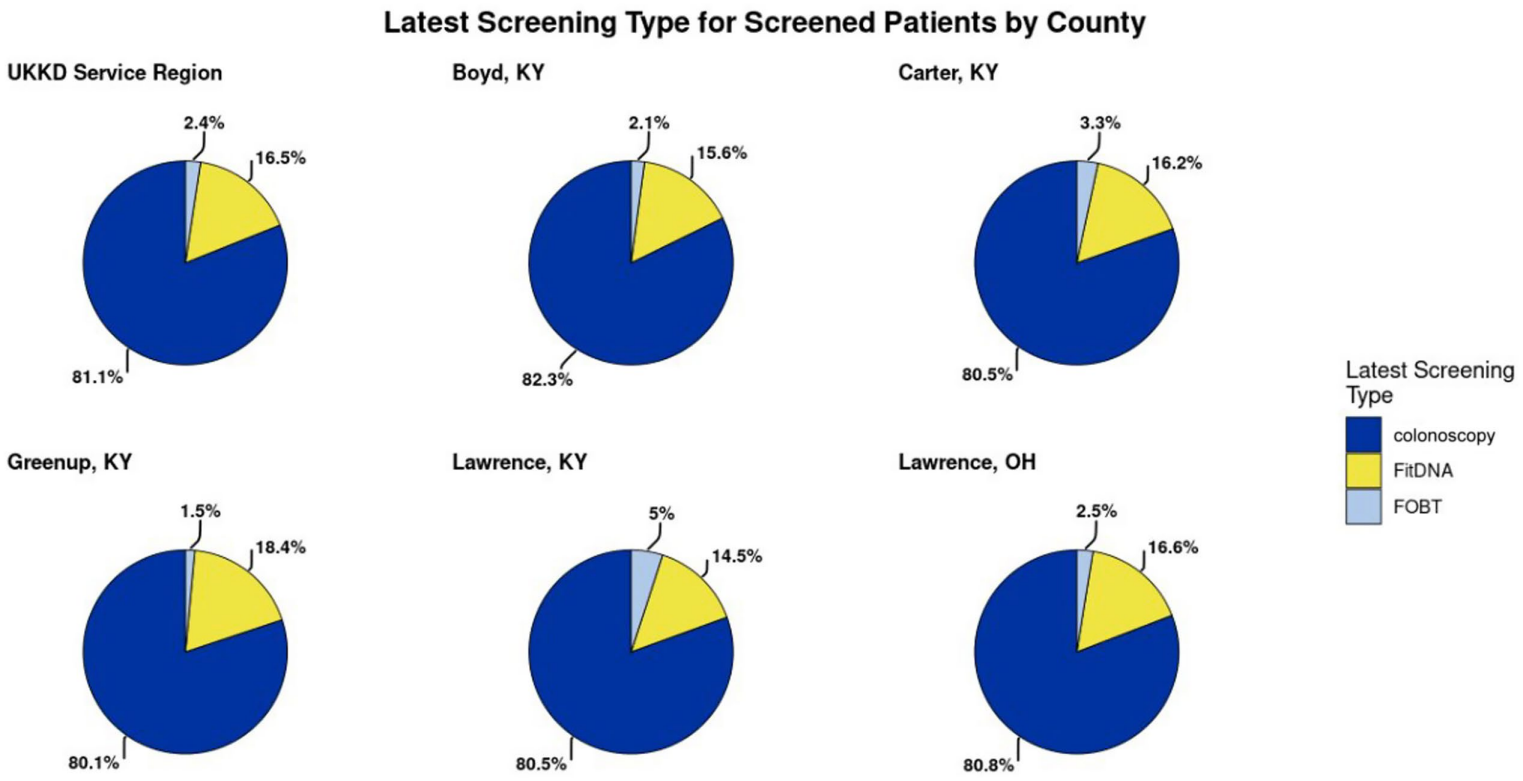
The distribution of the various types of screening methods for colorectal cancer. This was presented to the Action Team in order to inform members about the various screening methods.

Categorical variables are summarized in Table 2 by frequencies (N) and proportions (%), while continuous variables are summarized by means, standard deviations, medians, and first and third quartiles. Missing values were not included in the statistical analyses. Associations with cohorts and attributes were analyzed using Chi-Square tests for categorical variables and *t*-tests for continuous variables. A total of 22,549 individuals were included in the analysis presented in Table 3, with 7,756 in the not screened cohort and 14,793 in the screened cohort. The screened cohort was generally older, with a higher median age (60 vs. 57 years) and a greater proportion in the 50–75 age group (68.0% vs. 32.0%). The screened cohort also had a higher percentage of females (68.0% vs. 32.0%) compared to males (62.5% vs. 37.5%). Racial and ethnic composition varied between groups, though the majority identified as white, with smaller proportions of Black or African American, Hispanic, and other groups. Insurance status also differed: individuals in the screened cohort were more likely to have Medicare or commercial insurance, while the not screened cohort had higher percentages of Medicaid and self-pay patients. County of residence distributions showed that the screened cohort was more heavily represented across all service counties, with particularly high proportions from Boyd, Greenup, and Lawrence, KY. Clinic visit history differed between groups as well; the screened cohort had more individuals with frequent non-primary care (71.8%) and primary care (70.7%) clinic visits compared to the not screened cohort, which had higher proportions of individuals with no or fewer visits. As illustrated in Figure 1, more than 80% of CRC screenings across the UKKD service area were conducted via colonoscopy rather than low-prep, stool-based FOBT and FitDNA tests.

**Table 2:** Electronic health records (EHR) analytical findings.

Characteristic	*N* = 22,549
Age, years	
Mean (SD)	59.0 (8.2)
Median [Q1, Q3]	59.0 [52.0, 65.0]
Age groups	
45–50	3,498 (15.5%)
50–75	19,051 (84.5%)
Sex	
Female	12,781 (56.7%)
Male	9,768 (43.3%)
Race/ethnicity	
Black or African American	239 (1.1%)
Hispanic	36 (0.2%)
White	21,978 (97.5%)
Other	125 (0.6%)
Refused/unknown	171 (0.8%)
Insurance status	
Commercial	8,922 (39.6%)
Medicaid	3,799 (16.8%)
Medicare	9,111 (40.4%)
Other	145 (0.6%)
Self-pay	572 (2.5%)
County	
Boyd, KY	7,454 (33.1%)
Carter, KY	5,249 (23.3%)
Greenup, KY	5,122 (22.7%)
Lawrence, KY	812 (3.6%)
Lawrence, OH	3,912 (17.3%)

Q1, First Quartile; Q3, Third Quartile; SD, Standard Deviation. This table provides a summary of the colorectal cancer screening population within the 5 UKKD service counties.

**Table 3:** Comparison of demographic characteristics and healthcare utilization by colorectal cancer screening status.

Characteristic	Colorectal cancer screening			
	Overall	Not screened	Screened	*p*-value
	*N* = 22,549	*N* = 7,756	*N* = 14,793	
Age, years				<0.001
Mean (SD)	59.0 (8.2)	57.6 (8.4)	59.8 (8.1)	
Median [Q1, Q3]	59.0 [52.0, 65.0]	57.0 [51.0, 64.0]	60.0 [53.0, 66.0]	
Age groups				<0.001
45–50	3,498 (100.0%)	1,651 (47.2%)	1,847 (52.8%)	
50–75	19,051 (100.0%)	6,105 (32.0%)	12,946 (68.0%)	
Sex				<0.001
Female	12,781 (100.0%)	4,090 (32.0%)	8,691 (68.0%)	
Male	9,768 (100.0%)	3,666 (37.5%)	6,102 (62.5%)	
Race/ethnicity				<0.001
Black or African American	239 (100.0%)	65 (27.2%)	174 (72.8%)	
Hispanic	36 (100.0%)	18 (50.0%)	18 (50.0%)	
White	21,978 (100.0%)	7,543 (34.3%)	14,435 (65.7%)	
Other	125 (100.0%)	49 (39.2%)	76 (60.8%)	
Refused/unknown	171 (100.0%)	81 (47.4%)	90 (52.6%)	
Insurance status				<0.001
Commercial	8,922 (100.0%)	2,799 (31.4%)	6,123 (68.6%)	
Medicaid	3,799 (100.0%)	1,806 (47.5%)	1,993 (52.5%)	
Medicare	9,111 (100.0%)	2,740 (30.1%)	6,371 (69.9%)	
Other	145 (100.0%)	49 (33.8%)	96 (66.2%)	
Self-pay	572 (100.0%)	362 (63.3%)	210 (36.7%)	
County				<0.001
Boyd, KY	7,454 (100.0%)	2,386 (32.0%)	5,068 (68.0%)	
Carter, KY	5,249 (100.0%)	2,021 (38.5%)	3,228 (61.5%)	
Greenup, KY	5,122 (100.0%)	1,577 (30.8%)	3,545 (69.2%)	
Lawrence, KY	812 (100.0%)	293 (36.1%)	519 (63.9%)	
Lawrence, OH	3,912 (100.0%)	1,479 (37.8%)	2,433 (62.2%)	
Non-PCP clinic visits				<0.001
0	4,138 (100.0%)	2,077 (50.2%)	2,061 (49.8%)	
1	3,109 (100.0%)	1,357 (43.6%)	1,752 (56.4%)	
>1	15,301 (100.0%)	4,321 (28.2%)	10,980 (71.8%)	
Unknown	1	1	0	
PCP clinic visits				<0.001
0	2,993 (100.0%)	1,354 (45.2%)	1,639 (54.8%)	
1	5,293 (100.0%)	2,223 (42.0%)	3,070 (58.0%)	
>1	14,262 (100.0%)	4,178 (29.3%)	10,084 (70.7%)	
Unknown	1	1	0	

PCP, Primary Care Physician; Q1, First Quartile; Q3, Third Quartile; SD, Standard Deviation.

The principal barrier to timely EHR analysis was establishing the collaborative processes for this PPHA, specifically the time investment required to execute regulatory requirements such as the data use agreement and securing IRB approval. Gaining virtual machine access, in particular, involved multiple steps across various institutional personnel. While these security measures are essential for protecting patient data, the process could benefit from streamlining. Although regulatory requirements such as IRB approval, data use agreements, and virtual machine access were anticipated and integrated into our project timeline, the time and coordination required across institutional offices represented a key operational barrier. This experience not as a limitation of our model, but to inform other teams of the potential resource investment and to encourage early engagement with regulatory offices to prevent project delays. This aligns with findings that identified procedural inefficiencies and coordination challenges within institutions often contribute to delays in executing data use agreements, even when all parties are acting in good faith and with sufficient planning (35). Similarly, time and bureaucracy are common barriers to successful community engagement efforts, particularly in research involving multiple stakeholders (36).

Despite these delays, UKCPH team used the time productively to develop the SAP. This document served as a valuable guide for facilitating the pilot and future replications, facilitating focused discussions with the broader team about study aims and analytic strategies. As a result, once data access was granted, the team was able to proceed efficiently and conduct a high-quality analysis aligned with the original research objectives.

### 3.2 Inaugural PPHA action team meeting

**Figure 2:**
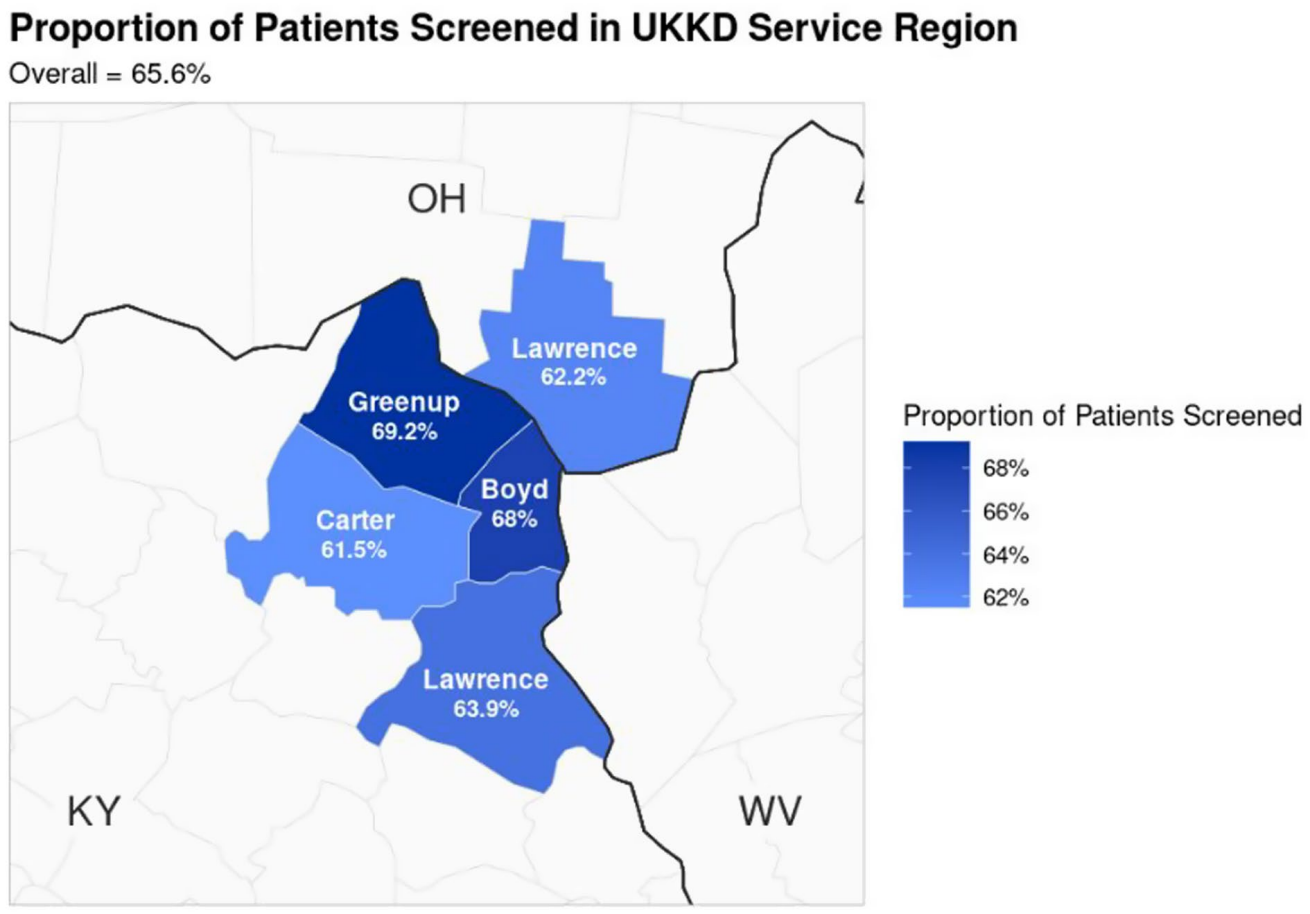
The proportion of UKKD patients screened for colorectal cancer. This was presented to the Action Team to show the distribution of patients screened within the service counties.

UKCPH researchers’ established relationships with Action Team members from previous studies facilitated successful recruitment and engagement in the inaugural PPHA meeting in March 2025. The first step of the meeting was UKCPH and UKKD presenting findings from analyses of secondary county-level data (Table 1) and EHR data (Tables 2, 3, Figures 1, 2). Then, UKKD described their current state in which responsibility for ensuring appropriate CRC screening had historically been on primary care and gastroenterologists. In March 2024, UKKD developed a physician-led, multidisciplinary CRC screening task force including all specialists to educate providers on CRC screening recommendations, implement EHR alerts for providers and multi-faceted contacts (calls, emails, patient portal) for patients overdue for screening, provide support for specialties that were not accustomed to involvement with screening, and strengthen patient outreach by offering stool-based testing at community events and partnership with the KY CancerLink to navigate financial burdens during the diagnosis and treatment process. Additionally, in the UKKD gastroenterology service line, the requirement for referrals to CRC screening was removed so that patients can self-refer and do not need to see a physician and are offered stool-based tests if colonoscopy is declined, with an instructional video for sample collection.

After receiving this information from UKCPH and UKKD, the Action Team discussed how this current state of UKKD CRC screening could be improved by increasing access to low-prep stool-based methods among younger eligible patients on Medicaid. An Action Team member representing the local health department and a community mental health center clinical manager attributed the lower rates of CRC screening among younger eligible patients on Medicaid to the logistical challenges of taking time off work for colonoscopy preparation and testing for lower-income workers. Additionally, an Action Team representative from the rape crisis center noted there may be trauma-related barriers to colonoscopy. After UKKD explained that colonoscopy is the gold standard for CRC screening, but Medicaid still covers FitDNA and FOBT for those for whom colonoscopy is not acceptable or appropriate, the Action Team prioritized increasing CRC screening among younger (aged 45–55) working-age patients on Medicaid with non-invasive methods to improve their communities’ CRC screening processes.

To meet these patients where they are in improving access to low-prep CRC screening, the Action Team co-designed a form for community partners to refer their patients and clients directly to UKKD. With this form, care coordinators at the health department and community mental health center (where Medicaid members receive a range of services) can directly schedule a colonoscopy for a patient or request a stool-based test be mailed to them. Nurse practitioners have established a call center to receive these referrals from community-based partners to order stool tests as well as follow up on results with the patients and referring agencies, and UKKD will track numbers of referrals received from each partner to report out at future meetings. UKKD also has provide FitDNA tests to community partners for distribution on-site to eligible patients.

Also in March 2025, UKKD presented findings to the UKKD CEO, physician leadership, and more than 200 physicians and advanced practitioners that patients with more than one encounter with both primary care and specialty providers were equally likely to complete CRC screening (Table 3). The presentation of this data assessing the importance of the UKKD CRC task force focus on coordinated CRC screening was a springboard for meaningful discussion about the power of holistic care and the value of collaboration between primary care providers and specialists. These initial convenings of the PPHA demonstrated that this approach is a promising solution for catalyzing, coordinating, and assessing context-specific practice improvements based on population-level analytics.

## 4 Discussion

Unlike technically oriented PPH approaches, the PPHA model and method for community engagement combines population health analytics, community-based intervention co-design, and clinical innovation to convene cross-sector partners and come to data-driven consensus on localized health improvement goals. The PPHA Action Team laid the groundwork for precision services and systems improvement interventions focused on additional priority areas in the region and provide a replicable model, method, and best practices for mobilizing multidisciplinary PPHAs in other communities. Furthermore, UKCPH’s thorough documentation of their data capture, cleaning, and analytical processes for EHR data enables us to apply the same approach to additional disease areas as well as share the SAP template with other teams.

However, as a pilot collaborative model, the replicability of the PPHA is predicated on collaboration with a healthcare system with sufficient EHR analytics and data-sharing capacity to engage with biostatisticians. Additionally, UKKD is the sole healthcare system serving these counties, which allows for a more complete representation of the population’s healthcare encounters than in regions with multiple healthcare enterprises. In areas with multiple providers, expanding collaboratives and agreements to include multiple partners can enhance data integration and representation. Additionally, utilizing state-level claims and discharge records can provide a broader and more inclusive dataset, addressing unmet social needs and structural risk factors effectively.

On a broader level, the PPHA model and methods hold great promise in both the analysis and interpretation of SDOH screening data and referral strategies (beyond the scope of the present study). Policies requiring the collection of social needs data will facilitate the addition of rich individual-level information on prevalent needs across the community (37). Analysis of these EHR data can help elucidate the relationships between critical health outcomes and social risk factors like food insecurity, housing instability, and safety. Results can then be used to inform clinical-community linkage strategies to ensure priority patient populations are being connected with community-based partners best equipped to meet their needs. Sharing integrated analyses of geographic, clinical, and social needs EHR data into Action Team meetings will provide a platform for examining the data within community-level contexts that can be used to inform policies and other relevant strategies that target the socioenvironmental factors influencing population health outcomes.

While the model and strategies presented here could improve upon the limitations of current PPH approaches that omit multidisciplinary engagement and quality improvement strategies, some limitations should be noted. As previously mentioned, ensuring healthcare partners have a robust EHR infrastructure that facilitates data collection and sharing is a core piece of the PPHA. Communities interested in replicating the PPHA model should carefully consider their roster of cross-sector partners as well as their patients and clients to ensure that integration into Action Teams is established early to build sustainable, trusting relationships. Organizational capacity also plays a critical role in commitment to the work, and some communities may struggle to develop and sustain the needed buy-in because of resource constraints. The one-year timeframe of this study limited our ability to assess the longer-term impacts of the alliance’s community engagement strategies. Future studies should apply systems evaluation strategies such as social network analysis (38) and Ripple Effects Mapping (39) to measure the extent to which alliance participation strengthened cross-sector partnership cohesion, perceived value, interconnectedness, and information-sharing. Considering how Action Team activities and data-driven findings can be used as advocacy tools can help communities establish funding and support streams that can build capacity across partners.

## Data Availability

The data analyzed in this study is subject to the following licenses/restrictions: The datasets are de-identified EHR records for a healthcare system that were only approved for internal quality improvement, not public dissemination. Access may be provided with Institutional Review Board determination. Requests to access these datasets should be directed to margaret.mcgladrey@uky.edu.
