# Supplementary material for: Precision public health alliances as a model and method for community engagement
Source: Front Public Health. 2025 Aug 21;13:1617776. doi: 10.3389/fpubh.2025.1617776 (PMC12408660; doi:10.3389/fpubh.2025.1617776)
Supplement: Supplementary file 1 [file Data_Sheet_1.docx]

**Statistical Analysis Plan (SAP)**

**Project Title:** Kentucky Precision Public Health Alliance Analysis of Social and Geo-Location Factors Associated with Gaps in Screenings and Other Prevention Indicators

**Project Number:**  CU17_0001

**Collaborative Unit Name (if applicable):**  UK King’s Daughters

**Investigators:**

**Primary Investigator:**  Dr. Charbel Salem

**Biostat CIRCL Biostatisticians:**  Dr. Kristen McQuerry, Kory Heier, Megan Hall, Caitlin

Phan

**Other Members:**  Dr. Beth Lacy, Dr. Rachel Graham, Dr. Margaret McGladrey, Dr.

Thomas Ard, Emily Clear, Dr. Svetla Slavova

**Original Creation Date:** 06Dec24

**Version:** 2.0

**Version Date:** 01May25

**Project Goal(s):**  Presentation of final analytic plan to UKKD and Action Team

**Submission Deadline(s):**  Dependent on EDC data access, goal is end of Feb 2025 to submit to CCTS Spring Conference (April 2025)

| **Investigator Agreement** | - All statistical analyses included in an abstract or manuscript will reflect the work of the biostatistician(s) listed on this SAP. No changes or additional analyses will be made to the results or findings without discussing with the project biostatistician(s). - All biostatisticians on this SAP will be given sufficient time to review the full presentation, abstract, manuscript, or grant and be included as co-authors on any abstract or manuscript resulting from the analyses. - If substantial additional analysis is necessary or the aims of the project change, a new SAP will need to be developed. - I have reviewed the SAP and understand that any changes must be documented.   Acknowledged by: Dr. Charbel Salem  Date: December 6, 2024 |
| --- | --- |

| **Activity Log** | 20Nov24: SAP creation  06Dec24: SAP v1 finalized  01May25: SAP v2 – Added Addendum Section 6 & updated analyses to reflect the addendum changes |
| --- | --- |

**1 Study Overview**

Background/Introduction: Precision public health leverages population-level data to inform decision-making in public health interventions tailored for communities. Kentucky has 120 counties that differ in geography, environment, health access, educational attainment, community-based organizations, school systems, demography, rurality, and regional affiliations. The pandemic taught us that a “one size fits all” population approach is ineffective and potentially harms public trust and partnerships. In Fall 2023, the UK College of Public Health (CPH) and the UK King’s Daughters (UKKD) medical center initiated a partnership to develop a precision public health approach focused on examining the interplay between social and clinical factors that underpin public health challenges specific to the region and populations served by UKKD.

Colorectal cancer represents eight percent of all new cancer cases in the United States. According to the National Cancer Institute, about 4.2 percent of men and women will be diagnosed with colorectal cancer at some point during their lifetimes. For most adults, older age is the most important risk factor for colorectal cancer, although being male and black are also associated with higher incidence and mortality. Colorectal cancer is most frequently diagnosed among people 65 to 74 years old. Rates of colorectal cancer in Kentucky are among the highest in the US.^1^

Screening can be effective for finding precancerous lesions (polyps) that could later become malignant, and for detecting early cancers that can be more easily and effectively treated. Precancerous polyps usually take about 10 to 15 years to develop into colorectal cancer, and most can be found and removed before turning into cancer. The five-year relative survival rate for people whose colorectal cancer is found in the early stage before it has spread is about 90 percent. The U.S. Preventive Services Task Force (2021) recommends screening for colorectal cancer in adults aged 45 to 49 years (Grade B recommendation) and in adults aged 50 to 75 years (Grade A recommendation; U.S. Preventive Services Task Force, 2021).

Colorectal cancer screening is a national quality measure. These measures are used by Medicare and Medicaid to assess the quality of care for beneficiaries, monitor performance at the state level, and improve the quality of health care.

**1.1 Study Aims**

What are the social and geo-location factors associated with gaps in colorectal cancer screening from November 2023-24.

**1.2 Research Questions**

Question 1: Are there associations with demographic factors and colorectal cancer screening completion?

Question 2: Are there associations with social determinants of health (SDoH) factors and colorectal cancer screening completion?

Question 3: Are there associations with clinical comorbidity factors and colorectal cancer screening completion?

**2 Study Population**

**2.1 Inclusion Criteria**

- Patients 46-75 years of age by the end of the November 2024 with a clinical visit between November 2023 and November 2024.

**2.2 Exclusion Criteria**

- Patients with a diagnosis or past history of total colectomy or colorectal cancer.
- Patients who were in hospice care or palliative care during the measurement year.
- Patients who meet frailty or dementia criteria.

**2.3 Data Acquisition**

| **Study design** | Retrospective Observational Study |
| --- | --- |
| **Data source/how the data were collected** | UKKD EDC |
| **Contact information for team member responsible for data collection/acquisition** | Ryan Parker; Ryan.Parker@kdmc.kdhs.us  Charbel Salem, Charbel.Salem@kdmc.kdhs.us |
| **Date or version (if downloaded, provide date)** | TBD |
| **Data transfer method and date** | UK sFTP |
| **Where the dataset is stored** | UK EDC Virtual Machine |
| **IRB number (if applicable)** | 99981 |

**3 Variables**

**3.1 Colorectal Screening Completion**

| **Variable Name** | **Description** | **Values** |
| --- | --- | --- |
| Denominator | Indicator variable for study population (patients 46-75 years of age by the end of the measurement period with a visit during the measurement period). All patients in the dataset should have a 1 for this variable. | 1 |
| Numerator | Indicator variable for colorectal cancer screening status. Patients were given a 1 (Screened) if they had an appropriate colorectal cancer screening, defined by  any one of the following criteria:   - Fecal occult blood test (FOBT) during the measurement period - Stool DNA (sDNA) with FIT during the measurement period or the two years prior to the measurement period - Flexible sigmoidoscopy during the measurement period or the four years prior to the measurement period - CT Colonography during the measurement period or the four years prior to the measurement period - Colonoscopy during the measurement period or the nine years prior to the measurement period | Binary:  0 = Not Screened  1 = Screened |
| Last_Colonoscopy | Date of the last colonoscopy recorded for the patient | Date |
| Last_Sigmoidoscopy | Date of the last sigmoidoscopy recorded for the patient | Date |
| Last_FitDNA | Date of the last Fit DNA recorded for the patient | Date |
| Last_FOBT | Date of the last Fecal Occult Blood Test (FOBT) recorded for the patient | Date |
| Last_Colonography | Date of the last colonography recorded for the patient | Date |
| latest_screening | *Derived variable:*  Date of last screening (any type) recorded for the patient | Date |
| latest_screening_type | *Derived variable:*  Type of last screening recorded for the patient. ‘Sigmoidoscopy’ and ‘Colonoscopy’ are grouped into *Colonoscopy.* | Categorical:  Colonoscopy  FitDNA  FOBT  Colonography |

**3.2 Demographic Factors**

| **Variable Name** | **Description** | **Values** |
| --- | --- | --- |
| Age | Patient’s age at beginning of measurement period | Numeric |
| Sex | Patient’s sex | Categorical:  F  M |
| Race_Ethnicity | *Recategorized:*  Patient’s race/ethnicity   - *Other* – ‘American Indian and Alaska Native’, ‘Asian’, ‘Vietnamese’, ‘Korean’, ‘Other’, ‘Asian Indian’, ‘Chinese’, ‘Native Hawaiian and Other Pacific Islander’, ‘Filipino’, ‘Japanese’ - *Refused/Unknown* – ‘Patient Refused’, ‘Unknown’ | Categorical:  White  Hispanic  Black or African American  Other  Refused/Unknown |
| Marital_Status | Patient’s marital status | Categorical:  Married  Divorced  Single  Significant Other  Widowed  Legally Separated  Other |
| Insurance_Status | *Recategorized:*  Patient’s insurance status   - *Commercial* – ‘Blue Cross’, ‘Commercial’ - Other – ‘Worker’s Comp’, ‘Tricare’, ‘Other’ | Categorical:  Medicaid  Self-pay  Commercial  Medicare  Other |
| KY_Medicaid | Indicator variable Medicaid status. Patients were given a 1 (Screened) if their primary coverage was a managed care Medicaid plans that counts towards the Medicaid Directed Payment Program | Binary:  0 = no Medicaid Directed Payment Program  1 = Medicaid Directed Payment Program |
| Zip_Code | Patient’s zip code | Numeric |
| County | Patient’s county. Only counties in the UKKD service region were included in the analysis. | Categorical:  BOYD  CARTER  GREENUP  LAWRENCE (KY)  LAWRENCE (OH) |
| State | Patient’s state | Categorical:  KY  OH |
| Rurality | *Derived variable:*  This variable will be derived based on HRSA^2^. | Categorical:  Not Fully FORHP Rural  Fully FORHP Rural |

**3.3 SDoH Factors**

| **Variable Name** | **Description** | **Values** |
| --- | --- | --- |
| SDOH_Transportation_Needs | SDOH Transportation Needs risk level domain | Categorical:  High Risk  Low Risk  Unknown |
| SDOH_Housing_ Stability | SDOH Housing Stability risk level domain | Categorical:  High Risk  Medium Risk  Low Risk  Unknown |
| SDOH_Intimate_Partner_Violence | SDOH Intimate Partner Violence risk level domain | Categorical:  High Risk  Low Risk  Unknown |
| SDOH_Food_Insecurity | SDOH Food Insecurity risk level domain | Categorical:  High Risk  Low Risk  Unknown |
| SDOH_Utilities | SDOH Utilities risk level domain | Categorical:  High Risk  Low Risk  Unknown |

**3.4 Clinical Factors**

| **Variable Name** | **Description** | **Values** |
| --- | --- | --- |
| Smoker | Indicator variable for patients that are a smoker during the measurement period and had not quite smoking prior to the measurement period | Binary:  0 = non-Smoker  1 = Smoker |
| Diabetes | Indicator variable for patients that are diagnosed with diabetes during the measurement period | Binary:  0 = no Diabetes  1 = Diabetes |
| Myocardial Infarction | Indicator variable for patients that are diagnosed with myocardial infarction during the measurement period | Binary:  0 = no Myocardial Infarction  1 = Myocardial Infarction |
| Angina | Indicator variable for patients that are diagnosed with angina during the measurement period | Binary:  0 = no Angina  1 = Angina |
| Stroke_TIA | Indicator variable for patients that are diagnosed with stroke or transient ischemic attack (TIA) during the measurement period | Binary:  0 = no Stroke/TIA  1 = Stroke/TIA |
| ASCVD | Indicator variable for patients that are diagnosed with atherosclerotic cardiovascular disease (ASCVD) during the measurement period | Binary:  0 = no ASCVD  1 = ASCVD |
| Ischemic | Indicator variable for patients that are diagnosed with ischemic heart disease during the measurement period | Binary:  0 = no Ischemic  1 = Ischemic |
| ESRD | Indicator variable for patients that are diagnosed with end-stage renal disease (ESRD) during the measurement period | Binary:  0 = no ESRD  1 = ESRD |
| Kidney_Transplant | Indicator variable for patients that are diagnosed with kidney transplant during the measurement period | Binary:  0 = no Kidney Transplant  1 = Kidney Transplant |
| CKD_Stage_5 | Indicator variable for patients that are diagnosed with chronic kidney disease (CKD) stage 5 during the measurement period | Binary:  0 = no CKD stage 5  1 = CKD stage 5 |
| Dialysis | Indicator variable for patients that are diagnosed with renal dialysis during the measurement period | Binary:  0 = no Dialysis  1 = Dialysis |
| Hepatitis | Indicator variable for patients that are diagnosed with hepatitis (acute or chronic) during the measurement period | Binary:  0 = no Hepatitis  1 = Hepatitis |
| Cirrhosis | Indicator variable for patients that are diagnosed with liver cirrhosis during the measurement period | Binary:  0 = no Cirrhosis  1 = Cirrhosis |
| COPD | Indicator variable for patients that are diagnosed with chronic obstructive pulmonary disease (COPD) during the measurement period | Binary:  0 = no COPD  1 = COPD |
| Asthma | Indicator variable for patients that are diagnosed with asthma (acute or chronic) during the measurement period | Binary:  0 = no Asthma  1 = Asthma |
| Cancer | Indicator variable for patients that are diagnosed with cancer during the measurement period | Binary:  0 = no Cancer  1 = Cancer |
| Obesity | Indicator variable for patients that are diagnosed with morbid or severe obesity during the measurement period | Binary:  0 = no Obesity  1 = Obesity |
| Anxiety | Indicator variable for patients that are diagnosed with anxiety during the measurement period | Binary:  0 = no Anxiety  1 = Anxiety |
| Depression | Indicator variable for patients that are diagnosed with any form of depression during the measurement period | Binary:  0 = no Depression  1 = Depression |
| Other_Mental_Health | Indicator variable for patients that are diagnosed with a mental health issue OTHER THAN anxiety or depression during the measurement period | Binary:  0 = no other Mental Health Illness  1 = Mental Health Illness |
| BMI_Most_Recent | Most recent BMI for the patient during the measurement period | Numeric |
| A1c_Mean | Mean of the patient’s A1c lab results during the measurement period | Numeric |
| A1c_Min | Minimum of the patient’s A1c lab results during the measurement period | Numeric |
| A1c_Max | Maximum of the patient’s A1c lab results during the measurement period | Numeric |
| A1c_Most_Recent | Most recent A1c lab result for the patient during the measurement period | Numeric |

**3.5 Visit Types**

| **Variable Name** | **Description** | **Values** |
| --- | --- | --- |
| Inpatient_Stays | Patient’s number of inpatient stays during the measurement period | Numeric |
| ER_Visits | Patient’s number of emergency room (ER) visits during the measurement period | Numeric |
| PCP_Clinic_Visits | *Recategorized:*  Patient’s number of primary care provider (PCP) visits during the measurement period. Recategorized from numeric to categorical. | Categorical:  0  1  >1 |
| Non_PCP_Clinic_Visits | *Recategorized:*  Patient’s number of non-primary care provider (PCP) visits during the measurement period. Recategorized from numeric to categorical. | Categorical:  0  1  >1 |

**3.6 Other**

| **Variable Name** | **Description** | **Values** |
| --- | --- | --- |
| Deidentified_PAT_ID | Unique patient identifier | Numeric |

**4 Statistical Analysis Plan**

Statistical analysis will be performed using R, version 4.4.1. Because this analysis requires multiple statistical tests, the Benjamini & Hochberg procedure will be used to adjust p-values.

**4.1 Analysis Plan for Research Question 1**

Table summaries and graphical displays of each demographic factor stratified colorectal screening completion will be presented. Overall (non-stratified) tables will also be included. Categorical variables will be summarized by frequencies (N) and proportions (%), while continuous variables will be summarized by means and standard errors. Associations with cohorts and attributes will be analyzed using Chi-Square tests for categorical variables and ANOVA for continuous variables. If cell counts for categorical variables are less than 5, Fisher’s Exact Test will be used. Parametric assumptions will be assessed prior to testing. Example tables can be found in Section 8. A heat map of county incidence rates of colorectal cancer screenings will be created.

**4.2 Analysis Plan for Research Question 2**

Table summaries and graphical displays of each SDoH factor stratified by colorectal screening completion will be presented. Analyses will be similar to Section 4.1. Example tables can be found in Section 8.

**4.3 Analysis Plan for Research Question 3**

Table summaries and graphical displays of each clinical comorbidity factor stratified by colorectal screening completion will be presented. Summaries will be presented separately for age groups 46-49 and 50-75. Example tables can be found in Section 8.

**5 Limitations**

Some variables within the data may have a substantial amount of missing data. Missing data may introduce bias, reduce statistical power, and limit the ability to draw reliable conclusions, especially if the missingness is not random.

**6** **Addendum for Additional Analyses**

For the Frontiers manuscript, statistical tests of t-test for continuous variables and chi squared for categorical variables were added to the table of demographic variables and clinic visits stratified by colorectal screening completion. Additionally, the two variables of high_med_risk and unknown were derived from the 5 SDOH variables of SDOH_Transportation_Needs, SDOH_Housing_Stability, SDOH_Intimate_Partner_Violence, SDOH_Food_Insecurity, and SDOH_Utilities. The high_med_risk variable indicates if a patient has at least one high risk or medium risk from the 5 SDOH variables. The unknown variable indicated if a patient has “Unknown” for all 5 of the SDOH variables.

**7 References**

1. <https://ecqi.healthit.gov/sites/default/files/ecqm/measures/CMS130v13.html>

2. <https://www.hrsa.gov/rural-health/about-us/what-is-rural/data-files>

3. <https://www.ncqa.org/hedis/measures/colorectal-cancer-screening/>

4. <https://www.ncqa.org/wp-content/uploads/2022/02/NCQA_COL_Resource_Guide_2022.pdf>

5. <https://www.cancer.org/content/dam/cancer-org/research/cancer-facts-and-statistics/colorectal-cancer-facts-and-figures/colorectal-cancer-facts-and-figures-2023.pdf>

**8 Appendix**

Table 1: Associations with Colorectal Cancer Screening Completion and Demographic Factors

|  | **Attribute** |  | **Completed Screening**  **N=XXX** | **Did Not Complete Screening**  **N=XXX** |
| --- | --- | --- | --- | --- |
| Demographic | Age | mean (stddev)[missing] | XX.XX (X.XXX) [XXX] | XX.XX (X.XXX) [XXX] |
|  |  | med (min, max) | XX.XX (XX.XX, XX.XX) | XX.XX (XX.XX, XX.XX) |
|  | Sex | Female | XXX (XX.X%) | XXX (XX.X%) |
|  |  | Male | XXX (XX.X%) | XXX (XX.X%) |
|  |  | Missing | XXX (XX.X%) | XXX (XX.X%) |
|  | Race/Ethnicity | White | XXX (XX.X%) | XXX (XX.X%) |
|  |  | Black | XXX (XX.X%) | XXX (XX.X%) |
|  |  | Hispanic | XXX (XX.X%) | XXX (XX.X%) |
|  |  | Asian or Pacific Islander | XXX (XX.X%) | XXX (XX.X%) |
|  |  | Native Americans | XXX (XX.X%) | XXX (XX.X%) |
|  |  | Missing | XXX (XX.X%) | XXX (XX.X%) |
|  | Marital Status | Single | XXX (XX.X%) | XXX (XX.X%) |
|  |  | Married | XXX (XX.X%) | XXX (XX.X%) |
|  |  | Divorced | XXX (XX.X%) | XXX (XX.X%) |
|  |  | Widowed | XXX (XX.X%) | XXX (XX.X%) |
|  |  | Missing | XXX (XX.X%) | XXX (XX.X%) |
|  | Insurance Type | XXXXX | XXX (XX.X%) | XXX (XX.X%) |
|  |  | …. |  |  |
|  | Zip Code | XXXXX | XXX (XX.X%) | XXX (XX.X%) |
|  |  | XXXXX | XXX (XX.X%) | XXX (XX.X%) |
|  |  | XXXXX | XXX (XX.X%) | XXX (XX.X%) |
|  |  | …. |  |  |
|  | County | XXXXX | XXX (XX.X%) | XXX (XX.X%) |
|  |  | …. |  |  |
|  | Rurality | XXXXX | XXX (XX.X%) | XXX (XX.X%) |
|  |  | …. |  |  |

Table 2: Associations with Colorectal Cancer Screening Completion and SDoH Factors

| **SDoH** | **Attribute** |  | **Completed Screening**  **N=XXX** | **Did Not Complete Screening**  **N=XXX** |
| --- | --- | --- | --- | --- |
| Transportation Challenges | Ability to Ensure Transportation to Healthcare Appointments | Yes | XXX (XX.X%) | XXX (XX.X%) |
|  |  | No | XXX (XX.X%) | XXX (XX.X%) |
|  |  | Missing | XXX (XX.X%) | XXX (XX.X%) |
| Nutrition Access Challenges | Food Insecurity | Yes | XXX (XX.X%) | XXX (XX.X%) |
|  |  | No | XXX (XX.X%) | XXX (XX.X%) |
|  |  | Missing | XXX (XX.X%) | XXX (XX.X%) |
|  | Access to Healthy Food | Yes | XXX (XX.X%) | XXX (XX.X%) |
|  |  | No | XXX (XX.X%) | XXX (XX.X%) |
|  |  | Missing | XXX (XX.X%) | XXX (XX.X%) |
| Household/Family Challenges | Domestic Violence | Yes | XXX (XX.X%) | XXX (XX.X%) |
|  |  | No | XXX (XX.X%) | XXX (XX.X%) |
|  |  | Missing | XXX (XX.X%) | XXX (XX.X%) |
|  | Safety Concerns at Home | Yes | XXX (XX.X%) | XXX (XX.X%) |
|  |  | No | XXX (XX.X%) | XXX (XX.X%) |
|  |  | Missing | XXX (XX.X%) | XXX (XX.X%) |
| Depression Screening | PHQ-2/PHQ-9 | mean (stddev)[missing] | XX.XX (X.XXX) [XXX] | XX.XX (X.XXX) [XXX] |
|  |  | med (min, max) | XX.XX (XX.XX, XX.XX) | XX.XX (XX.XX, XX.XX) |
| Financial Instability | Issues Paying for Medications, Utilities, or Healthcare | Yes | XXX (XX.X%) | XXX (XX.X%) |
|  |  | No | XXX (XX.X%) | XXX (XX.X%) |
|  |  | Missing | XXX (XX.X%) | XXX (XX.X%) |
| Housing Instability | Homeless | Yes | XXX (XX.X%) | XXX (XX.X%) |
|  |  | No | XXX (XX.X%) | XXX (XX.X%) |
|  |  | Missing | XXX (XX.X%) | XXX (XX.X%) |
|  | Frequent Moves | Yes | XXX (XX.X%) | XXX (XX.X%) |
|  |  | No | XXX (XX.X%) | XXX (XX.X%) |
|  |  | Missing | XXX (XX.X%) | XXX (XX.X%) |

Table 3: Associations with Colorectal Cancer Screening Completion and Clinical Comorbidities Factors

| **Chronic Conditions** | **Attribute** |  | **Completed Screening**  **N=XXX** | **Did Not Complete Screening**  **N=XXX** |
| --- | --- | --- | --- | --- |
| Diabetes | HbA1c levels | mean (stddev)[missing] | XX.XX (X.XXX) [XX] | XX.XX (X.XXX) [XX] |
|  |  | med (min, max) | XX.XX (XX.XX, XX.XX) | XX.XX (XX.XX, XX.XX) |
|  | Presence | Type 1 | XXX (XX.X%) | XXX (XX.X%) |
|  |  | Type 2 | XXX (XX.X%) | XXX (XX.X%) |
|  |  | Missing | XXX (XX.X%) | XXX (XX.X%) |
| Cardiovascular Conditions | History of Heart Failure, Hypertension, Coronary Artery Disease | Yes | XXX (XX.X%) | XXX (XX.X%) |
|  |  | No | XXX (XX.X%) | XXX (XX.X%) |
|  |  | Missing | XXX (XX.X%) | XXX (XX.X%) |
| Renal Conditions | Chronic Kidney Disease Stage | 1 | XXX (XX.X%) | XXX (XX.X%) |
|  |  | 2 | XXX (XX.X%) | XXX (XX.X%) |
|  |  | 3 | XXX (XX.X%) | XXX (XX.X%) |
|  |  | 4 | XXX (XX.X%) | XXX (XX.X%) |
|  |  | 5 | XXX (XX.X%) | XXX (XX.X%) |
|  | Dialysis Requirement | Yes | XXX (XX.X%) | XXX (XX.X%) |
|  |  | No | XXX (XX.X%) | XXX (XX.X%) |
|  |  | Missing | XXX (XX.X%) | XXX (XX.X%) |
| Liver Diseases | Cirrhosis | Yes | XXX (XX.X%) | XXX (XX.X%) |
|  |  | No | XXX (XX.X%) | XXX (XX.X%) |
|  |  | Missing | XXX (XX.X%) | XXX (XX.X%) |
|  | Hepatitis | Yes | XXX (XX.X%) | XXX (XX.X%) |
|  |  | No | XXX (XX.X%) | XXX (XX.X%) |
|  |  | Missing | XXX (XX.X%) | XXX (XX.X%) |
| Lung Diseases | COPD | Yes | XXX (XX.X%) | XXX (XX.X%) |
|  |  | No | XXX (XX.X%) | XXX (XX.X%) |
|  |  | Missing | XXX (XX.X%) | XXX (XX.X%) |
|  | Asthma | Yes | XXX (XX.X%) | XXX (XX.X%) |
|  |  | No | XXX (XX.X%) | XXX (XX.X%) |
|  |  | Missing | XXX (XX.X%) | XXX (XX.X%) |
| Other Chronic Conditions | Cancer | Yes | XXX (XX.X%) | XXX (XX.X%) |
|  |  | No | XXX (XX.X%) | XXX (XX.X%) |
|  |  | Missing | XXX (XX.X%) | XXX (XX.X%) |
|  | Obesity | Yes | XXX (XX.X%) | XXX (XX.X%) |
|  |  | No | XXX (XX.X%) | XXX (XX.X%) |
|  |  | Missing | XXX (XX.X%) | XXX (XX.X%) |
|  | Mental Health Disorders | Yes | XXX (XX.X%) | XXX (XX.X%) |
|  |  | No | XXX (XX.X%) | XXX (XX.X%) |
|  |  | Missing | XXX (XX.X%) | XXX (XX.X%) |
